# The clinicopathological and prognostic features of Chinese and Japanese inpatients with lung cancer

**DOI:** 10.18632/oncotarget.11850

**Published:** 2016-09-06

**Authors:** Yang Gao, Ji-feng Zhang, Qing-chang Li, Jia-jie Liu, Li-li Liu, Xue-feng Yang, Hua-mao Jiang, Hua-chuan Zheng

**Affiliations:** ^1^ Cancer Center, Key Laboratory of Brain and Spinal Cord Injury of Liaoning Province, and Animal Center, The First Affiliated Hospital of Jinzhou Medical University, Jinzhou 121001, China; ^2^ Department of Pathology, The First Affiliated Hospital of China Medical University, Shenyang 110001, China; ^3^ Department of Urology, The First Affiliated Hospital of Jinzhou Medical University, Jinzhou 121001, China; ^4^ Life Science Institute of Jinzhou Medical University, Jinzhou 121001, China

**Keywords:** lung cancer, clinicopathological behaviors, prognosis, China, Japan

## Abstract

Here, we retrospectively compared the differences in clinicopathological behaviors and prognosis of lung cancer from the First Affiliated Hospital (CMU1, n=513), Shengjing Hospital (CMUS, n=1021), Tumor Hospital (CMUT, n=5378) of China Medical University, the First Affiliated Hospital of Dalian (DMU, n=2251) and Jinzhou (JMU, n=630) Medical University, Takaoka Kouseiren Hospital (Takaoka, n=163) of Japan. Japanese lung cancer patients showed smaller tumor size, lower TNM staging, lower ratio of squamous cell carcinoma and higher ratio of small and large cell carcinomas than Chinese patients (p<0.05). Survival analysis showed that tumor size was employed as a prognostic factor for the Japanese and Chinese cancer patients (p<0.05). In DMU and CMUS, the ratios of female patients or adenocarcinoma were higher than other hospitals (p<0.05), while the patients from CMUT and CMU1 were younger than the others (p<0.05). The ratios of squamous cell carcinoma from CMUT, CMU1 and JMU were higher than the others, while it was the same for the ratio of large and small cell carcinoma in Takaoka and CMU1 (p<0.05). TNM staging was higher in CMUT than JMU and Takaoka (p<0.05). The female patients of lung cancer showed young prone, large tumor size, a high ratio of adenocarcinoma and advanced TNM staging in comparison to the counterpart (p<0.05). The younger patients of lung cancer displayed smaller tumor size, higher ratio of adenocarcinoma, lower TNM staging than the elder in Takaoka (p<0.05). There were more aggressive behaviors and shorter survival time for Chinese than Japanese lung cancer patients. The prevention of lung cancer should be strengthened by establishing a systematic and effective screening strategy, especially for the young and female patients.

## INTRODUCTION

At present, lung cancer is one of the malignant tumors with the highest morbidity and mortality rates and remains a major public health problem worldwide. In 2008, there were an estimated 1.61 million of new cases, representing 12.7% of all new cancers. Lung cancer was the most common cause of cancer-related death with 1.38 million deaths (18.2% of the total) worldwide [1]. Although lung cancer death rates are decreasing in most Western countries, lung cancer shows an increasing incidence rate [2, 3], is still the most common cancer and the leading cause of cancer-related deaths in China [4].

Lung cancer was the first common cancer in Chinese males and the second after breast cancer in Chinese females [5]. According to a recent study, there were total 605,946 new cases of lung cancer in 2010, including 416,333 (68.7%) men and 189,613 (31.3%) women [4]. The incidence ratio of lung cancer between males and females was 2.21, which might decrease in the next few years because lung cancer incidence in women was increasing faster than that in men from 1988 to 2005 (1.3% in men and 2.34% in women) [3]. With a large smoking population, the growth of lung cancer incidence continues to rise in China [6]. According to the data from National Central Cancer Registry [5], the average age of lung cancer incidence among male and female dramatically increased from 65.32 and 65.14 to 67.87 and 68.05 years old during 1989-2008 respectively [7]. In the recent years, adenocarcinoma has replaced squamous cell carcinoma as the most predominant histological sub-type of lung cancer in China, which is consistent with the change in developed countries [8, 9]. Reportedly, the frequencies of adenocarcinoma increased from 21.96% to 43.4% and frequencies of squamous cell carcinoma decreased from 39.11% to 32.23% in 15,427 male lung cancer patients during 2003-2013 [10].

In this paper, we reported the clinicopathological characteristics of lung cancers from The First Affiliated Hospital (CMU1), Shengjing Hospital (CMUS), Tumor Hospital (CMUT) of China Medical University, The First Affiliated Hospital of Dalian (DMU) and Jinzhou (JMU) Medical University, Takaoka Kouseiren Hospital (Takaoka) of Japan at aim to find out the prevention strategy for lung cancer in China.

## RESULTS

**Figure 1:**
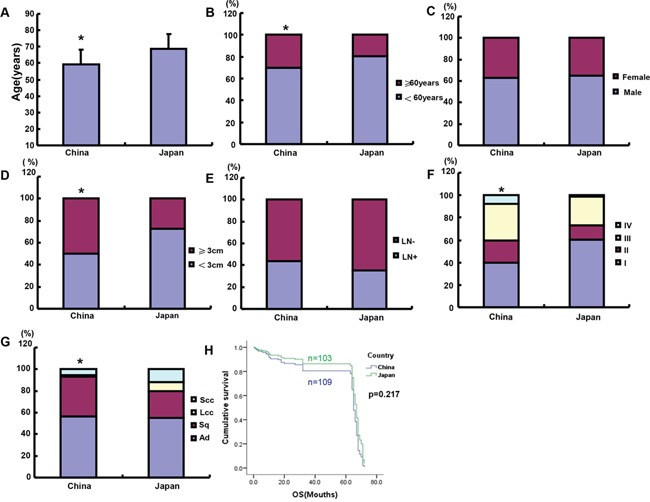
The clinicopathological features of Chinese and Japanese patients with lung cancer The parameters of Chinese and Japanese lung cancer patients were compared, including age **A, B.** gender **C.** tumor size **D.** lymph node metastasis **E.** TNM staging **F.** histological subtyping **G.** and survival time **H.** LN, lymph node metastasis; Ad, adenocarcinoma; Sq, squamous cell carcinoma; LCC, large cell carcinoma; SCC, small cell carcinoma; *p<0.05, compared with Japanese patients.

As shown in Table 1 and Figure 1A, the mean age of Chinese lung cancer patients was 59.25 ± 9.83 years old, significantly lower than that of Japanese ones, which had an age of 68.77 ± 8.82 years old (n=162, Table 1, p<0.05), indicating that the elder patients with CRC was diagnosed in Japan than China (Figure 1B). The mean age was 59.55 ± 9.74 years old for the male patients and 58.76 ± 9.96 years old for the female patients in China. The mean ages were 69 ± 7.88 and 66.78 ± 10.45 years old for the male and female patients in Japan respectively. Lung cancer more frequently occurred in male than in female patients of both the countries (Table 1, p<0.05). The ratios of lung cancer between male and female were 1.7 and 2.1 in China and Japan respectively, while no significant difference was observed in both countries (Figure 1C). Lung cancers exhibited larger tumor size and higher TNM staging in Chinese than Japanese patients, irrespective of gender (Figure 1D and 1F, p<0.05). No difference in lymph node metastasis was found between Chinese and Japanese patients with CRC (Figure 1E, p<0.05). Japanese female group had an early stage, compared to male group (Table 1, p<0.05). Clinicopathological staging of lung cancer patients was earlier in Japan than China (Figure 1F, p<0.05). Staging-IV lung cancer was more frequently observed in Chinese than Japanese patients (Table 1, p<0.05). The most histological type was adenocarcinoma (Ad), followed by squamous cell carcinoma (Sq) in both the countries. The proportion of large or small cell carcinomas was larger in Japan than that in China (Figure 1G, p<0.05), while versa for Sq (Figure 1G, p<0.05). Sq was the most frequent in the male group in both China and Japan, while Ad was still the most frequent in female group, accounting for more than 80%. As shown in Table 2, the elder patients (≧50 years) were more prevalent than the counterpart in both the countries (p<0.05). The high-incidence age was 51-60 years old, responsible for 37.1% and 37.7% of male and female cases in China. In Japan, the incidence increased with the age, reaching the peak at the age of 71-80 years old.

**Table 1:** Clinicopathological features of Chinese and Japanese patients with lung cancer

Clinicopathological features	n	China	n	Japan
Age(mean +SD, yr)	9793	59.25+9.83*	162	68.77+8.82
Sex(male : female)	9776	6151:3625*	154	104:50
Tumor size(<3cm:≥3cm)	2132	1062:1070*	99	72:27
Lymph node metastasis(+)	2324	1013(43.6) *	153	54(35.3)
Histological type	8948		156	
Adenocarcinoma		4910(54.9)		86(55.1)
Squamous cell carcinoma		3270(36.5) *		38(24.4)
Small cell carcinoma		494(5.5) *		18(9.0)
Large cell carcinoma		274(3.1) *		14(11.5)
TNM staging	2608		101	
I		1030(39.49) *		61(60.4)
II		533(20.4)		13(12.9)
III		846(32.4)		26(25.7)
IV		199(7.6) *		1(1.0)

* p<0.05, compared with Japanese patients

**Table 2:** The relationship between age and gender of Chinese and Japanese patients with lung cancer

Age (years)	China, n (%)			Japan, n (%)		
	Male	Female	Total	Male	Female	Total
<30	22(0.4)	18(0.5)	40(4.1) *	0(0.0)	0(0.0)	0(0.0)
31-40	153(2.5)	118(3.3)	271(2.8) *	0(0.0)	1(2.0)	1(0.7)
41-50	884(14.4)	567(15.6)	1451(14.8) *	1(1.0)	1(2.0)	2(1.3)
51-60	2281(37.1)	1349(37.7)	3630(37.1)*	18(17.3)	15(30.0)	33(21.4)
61-70	1929(31.4)	1113(30.7)	3042(31.1)	33(31.7)	14(28.0)	47(30.5)
71-80	815(13.2)	443(12.2)	1258(12.9) *	49(47.1)	15(30.0)	64(41.6)
>80	67(1.1)	17(0.5)	84(8.6)	3(2.9)	4(8.0)	7(4.6)
	6151(62.9)	3625(37.1)		104(67.5)	50(32.5)	

* p<0.05 compared with Japanese lung cancer patients

### Comparison of the prognosis of Japanese and Chinese patients with lung cancer

We followed up Chinese and Japanese patients with lung cancer and established Kaplan-Meier curves (Figure 1H). Logistic rank test showed that the survival rate of Chinese patients was lower than that of Japanese ones, but no significance was found (p>0.05). The univariate analysis showed that only tumor size was a prognostic factor for both Japanese and Chinese patients with lung cancer (Table 3, p<0.05). It was the same for Chinese patients (Table 4, p<0.05). However, all the parameters except lymph node metastasis were significantly correlated with a long survival time, including male, the old age, small tumor, low TNM staging, and histological subtyping for Japanese patients (Table 4, p<0.05). Multivariate analysis showed that both tumor size and lymph node metastasis were independent factors to indicate the prognosis of lung cancer (Table 5, p<0.05). Although there was no independent prognostic factors for Chinese patients, tumor size, TNM staging and lymph node metastasis were independent for Japanese ones (Table 6, p<0.05).

**Table 3:** Univariate analysis of clinicopathological variables for the survival of the patients with lung cancer

Parameters	95.0% CI for Exp(B)	p value
Country	0.642(0.317-1.298)	0.217
Sex	1.101(0.666-1.820)	0.706
Age	1.044(0.668-1.632)	0.850
Tumor size	0.430(0.266-0.696)	0.001
Lymph node metastasis	0.647(0.382-1.096)	0.105
TNM staging	1.483(0.734-2.996)	0.272
Histological subtyping	1.289(0.831-1.998)	0.257

CI=confidence interval

**Table 4:** Univariate analysis of clinicopathological variables for the survival of the Chinese and Japanese patients with lung cancer

Parameters	China		Japan	
	95.0% CI for Exp(B)	p value	95.0% CI for Exp(B)	p value
Sex	1.319(0.761-2.286)	0.324	14.122(1.511-132.014)	0.020
Age	0.941(0.590-1.500)	0.798	0.048(0.004-0.522)	0.013
Tumor size	0.496(0.285-0.864)	0.013	0.001(0.000-0.064)	0.001
Lymph node metastasis	0.620(0.344-1.117)	0.111	0.359(0.078-1.654)	0.189
TNM staging	1.902(0.762-4.751)	0.168	0.049(0.006-0.398)	0.005
Histological subtyping	1.077(0.666-1.740)	0.764	894.901(16.228-49349.809)	0.001

CI=confidence interval

**Table 5:** Multivariate analysis of clinicopathological variables for the survival of the patients with lung cancer

Parameters	95.0% CI for Exp(B)	p value
Country	1.603(0.722-3.559)	0.246
Sex	0.811(0.506-1.300)	0.384
Age	0.981(0.632-1.524)	0.934
Tumor size	2.158(1.364-3.416)	0.001
Lymph node metastasis	1.619(1.020-2.570)	0.041
TNM staging	0.833(0.520-1.334)	0.446
Histological subtyping	0.874(0.604-1.264)	0.474

CI=confidence interval

**Table 6:** Multivariate analysis of clinicopathological variables for the survival of Chinese and Japanese patients with lung cancer

Parameters	China		Japan	
	95.0% CI for Exp(B)	p value	95.0% CI for Exp(B)	p value
Sex	0.437(1.314-3.478)	0.758	1.057(0.321-0.324)	0.928
Age	0.667(1.694-6.544)	1.063	1.575(0.379-0.798)	0.532
Tumor size	1.158(3.512-24.733)	2.016	7.218(2.106-0.013)	0.002
Lymph node metastasis	0.896(2.903-9.699)	1.612	3.615(1.348-0.111)	0.011
TNM staging	0.210(1.313-3.304)	0.526	1.818(1.000-0.168)	0.049
Histological subtyping	0.575(1.502-1.276)	0.929	0.632(0.313-0.764)	0.201

CI=confidence interval

### Clinicopathological features of the lung cancer patients of different ages and genders

**Figure 2:**
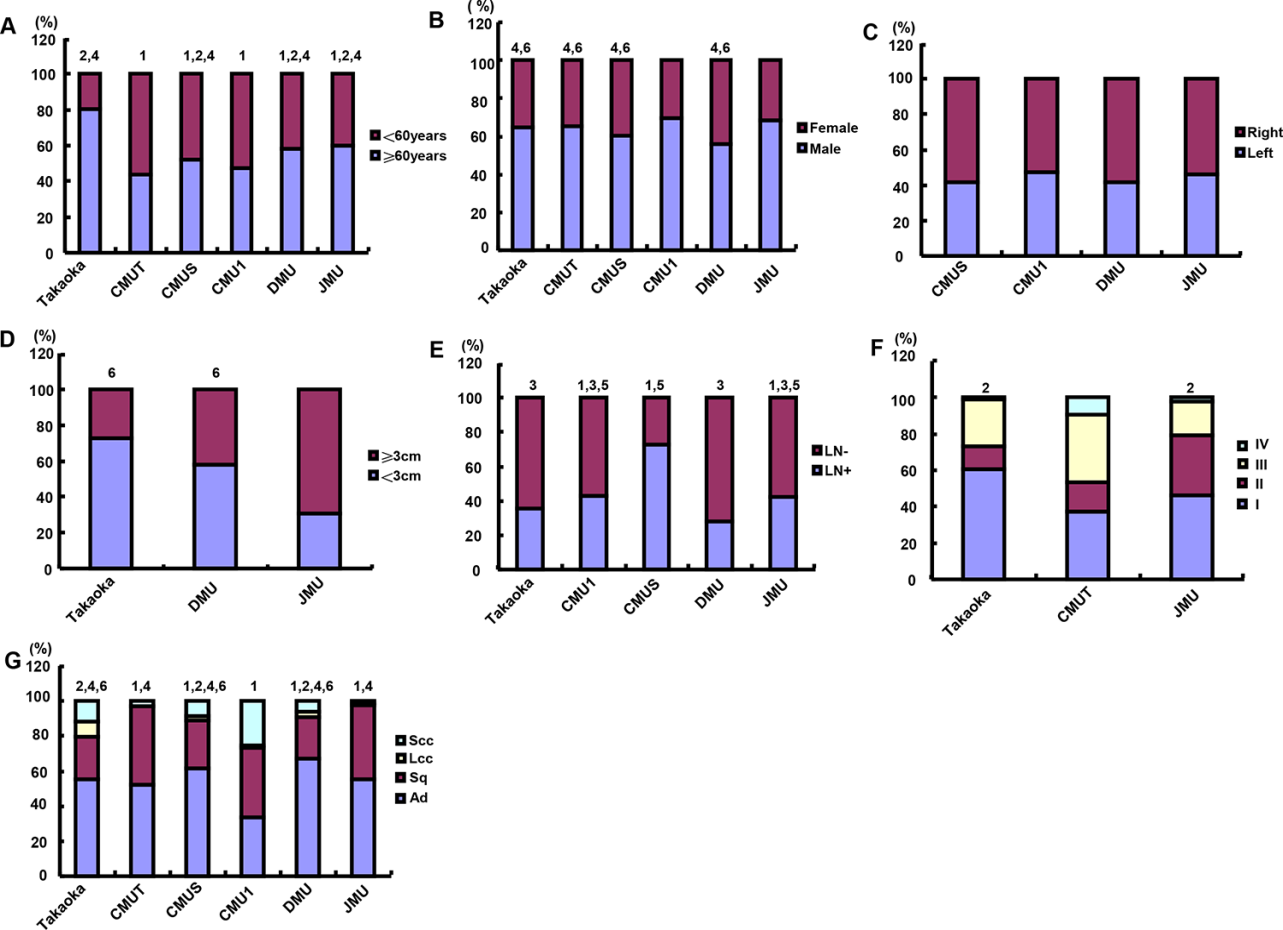
The differences in clinicopathological features of the patients with lung cancer from different hospitals The differences in age **A.** gender **B.** tumor localization **C.** tumor size **D.** lymph node metastasis **E.** TNM staging **F.** histological subtyping **G.** was analyzed for the lung cancer patients from different hospitals. Note: CMU1, The First Affiliated Hospital of China Medical University; CMUS, Shengjing Hospital of China Medical University; CMUT, Tumor Hospital of China Medical University; DMU, The First Affiliated Hospital of Dalian Medical University; JMU, The First Affiliated Hospital of Jinzhou Medical University; Takaoka, Takaoka Kouseiren Hospital of Japan; LN, lymph node metastasis; Ad, adenocarcinoma; Sq, squamous cell carcinoma; LCC, large cell carcinoma; SCC, small cell carcinoma; (1, p<0.05 vs Takaoka; 2, p<0.05 vs CMUT; 3, p<0.05 vs CMUS; 4, p<0.05 vs CMU1; 5 p<0.05 vs DMU; 6, p<0.05 vs JMU).

The patients from CMUT and CMU1 were younger than the other hospitals, while the patients from Takaoka were eldest in the 6 hospitals (Figure 2A, p<0.05). As indicated in Figure 2B, the proportion of male patients was comparatively higher in CMU1 and JMU than the other hospitals (p<0.05). There was no difference in tumor localization between these hospitals (p>0.05), but the right cancers were more frequently observed than the left (Figure 2C). Tumor size was gradually decreasing from Takaoka, DMU to JMU, while TNM staging advanced from Takaoka, JMU to CMUT (Figure 2D and 2F, p<0.05). The occurrence rate of lymph node metastasis was lower in Takaoka and DMU than others, but highest in CMUS (Figure 2E, p<0.05). There was the lowest ratio of adenocarcinoma, while the highest rate of Sq in CMUT, CMU1 and JMU (Figure 2G, p<0.05). Higher ratios of small and large cell carcinomas were observed in Takaoka and CMU1 than the other hospitals (Figure 2G, p<0.05).

**Figure 3:**
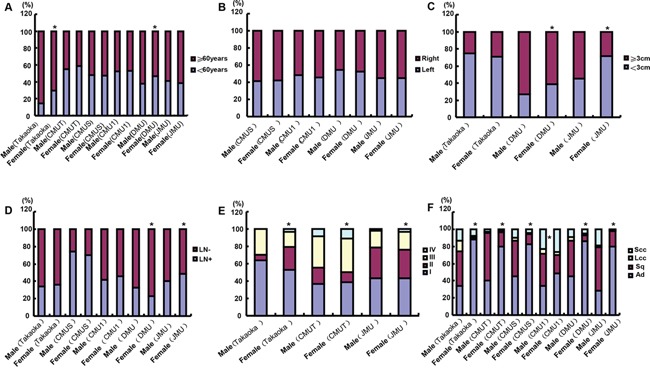
The clinicopathological features of the lung cancer patients of different genders According to the gender stratification, we compared the differences in age **A.** tumor localization **B.** tumor size **C.** lymph node metastasis **D.** TNM staging **E.** histological subtyping **F.** was analyzed for the lung cancer patients from different hospitals. Note: CMU1, The First Affiliated Hospital of China Medical University; CMUS, Shengjing Hospital of China Medical University; CMUT, Tumor Hospital of China Medical University; DMU, the First Affiliated Hospital of Dalian Medical University; JMU, the First Affiliated Hospital of Jinzhou Medical University; Takaoka, Takaoka Kouseiren Hospital of Japan; LN, lymph node metastasis; Ad, adenocarcinoma; Sq, squamous cell carcinoma; LCC, large cell carcinoma; SCC, small cell carcinoma; *p<0.05, compared with male patients.

The female patients were younger than the male in Takaoka and DMU (Figure 3A, p<0.05). No difference was observed in tumor localization of lung cancers between male and female patients of different hospitals (Figure 3B, p>0.05). Tumor size was larger in the female than male patients of DMU and JMU (Figure 3C, p<0.05). Female patients had a lower ratio of lymph node metastasis than the male ones in DMU, while versa for JMU (Figure 3D, p<0.05). TNM staging was more advanced in the female than male patients of CMUT and JMU (Figure 3E, p<0.05). The ratio of Ad was higher in female patients of Takaoka, CMUT, CMUS, CMU1, DMU and JMU than the male ones (Figure 3F, p<0.05).

**Figure 4:**
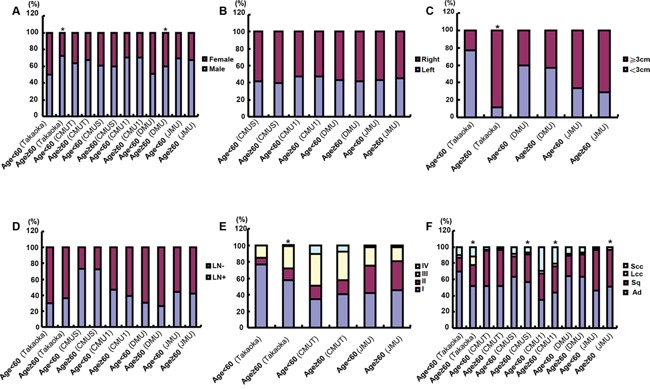
The clinicopathological features of the lung cancer patients of different ages According to the age stratification, we compared the differences in gender **A.** tumor localization **B.** tumor size **C.** lymph node metastasis **D.** TNM staging **E.** histological subtyping **F.** was analyzed for the lung cancer patients from different hospitals. Note: CMU1, The First Affiliated Hospital of China Medical University; CMUS, Shengjing Hospital of China Medical University; CMUT, Tumor Hospital of China Medical University; DMU, The First Affiliated Hospital of Dalian Medical University; JMU, The First Affiliated Hospital of Jinzhou Medical University; Takaoka, Takaoka Kouseiren Hospital of Japan; LN, lymph node metastasis; Ad, adenocarcinoma; Sq, squamous cell carcinoma; LCC, large cell carcinoma; SCC, small cell carcinoma; *p<0.05, compared with younger patients (<60 years).

As shown in Figure 4A, the younger patients were more female than the elder in Takaoka and DMU (p<0.05). There was no association of age with tumor localization or lymph node metastasis in the present study (Figure 4B and 4D, p>0.05). In Takaoka, the tumor size was larger in the elder than younger patients (Figure 4C, p<0.05). In Takaoka, lung cancers showed advanced TNM staging in elder patients than younger patients (Figure 4E, p<0.05). Small and large cell carcinoma were less frequently observed in younger than elder patients in Takaoka and JMU, while versa in CMUS and CMU1 (SCC, Figure 4F, p<0.05). In CMUS and Takaoka, the elder patients more suffered from Sq and less Ad than younger ones (Figure 4F, p<0.05), while the converse was true for JMU (Figure 4F, p<0.05). In CMU1, Ad was less diagnosed in younger than elder patients (Figure 4F, p<0.05), while versa for SCC (Figure 4F, p<0.05).

## DISCUSSION

Lung cancer is the deadliest cancer worldwide, whose incidence is increasing in Asia, especially high in men [11]. In comparison to Chinese patients, we found that Japanese lung cancer patients exhibited small tumor size, low TNM staging, low ratio of squamous cell carcinoma and high ratio of small and large cell carcinomas, suggesting that more aggressiveness of lung cancer in China than Japan. In both countries, the ratio of male to female inpatients was 3: 2, compared to 2.21 of all China in 2010 [2]. This might be due to more exposure to occupational risk, smoking and mental burden for men. In contrast, a study have demonstrated that lung cancer incidence rate in women is increasing faster than that in men [3]. It was found that the average age of lung cancer patients was approximately 10 years younger in Chinese than Japanese patients. In China, we chose the hospitals of Liaoning province, where rapid economic development had also inevitably brought serious environmental pollution due to coal burning, waste gas discharge and vehicle emissions [12]. The average PM2.5 exposure concentration was higher in China than Japan, regardless of cooking + eating, heating or illumination [13]. In Japan, improved tumor screening system had been established for many decades, such as wide implementation of low-dose CT screening for citizen older than 40 years old, but not yet due to low economic condition in China, where many lung cancer patients are diagnosed firstly in advanced stage and with a larger tumor size. High smoking rate is seen in the northeastern of China and is very common in women due to the habits of Man ethnicity, especially in Jinzhou. Reportedly, the lung cancer risk associated with smoking was greater for Japanese than for Chinese women after controlling for age and socioeconomic index [14]. It was the same for the relative risk of friends’ smoking for students [15]. The right lung cancers more frequently occurred than the left, which might be attributed to the involvement of their anatomical features in susceptibility to carcinogens because the right main bronchus is thick and short. It was also supported by a higher positive rate of Epstein-Barr virus infection in right lung cancer [16].

In the present study, the shorter survival time of Chinese patients than Japanese ones might result from the difference in aggressiveness. Additionally, the standard protocol of Japanese Cancer Society for the dissection of lung cancer is strictly carried out in Japan, which also improves the survival of Japanese lung cancer patients. Tumor size was employed as a prognostic factor for the Japanese and Chinese patients with lung cancer regardless of univariate and multivariate analysis. Zhang et al. [17] found that tumor size significantly influenced cumulative survival time of non-small cell lung cancers after adjusting for lymph node examination and disease extension. The data from a Japanese investigator showed that non-Ad histology, elevated serum CEA at recurrence and no systemic chemotherapy were independent unfavorable post-recurrence prognostic factors for lung cancers [18] although prognostic factors of lung cancer also included female sex and 70 years of age or younger [19]. Jiang et al. [20] reported that Glasgow Prognostic Score (GPS) was more accurate than prognostic index in predicting prognosis for patients with advanced NSCLC, but GPS only can be used for preliminary assessment because of low predicting accuracy.

In China, the five hospitals of Liaoning Province were enrolled in the present study, among which DMU and JMU were localized near to the sea, while the other three in the provincial capital. In DMU and CMUS, the ratios of female patients and Ad were higher than other hospitals, while the patients from CMUT and CMU1 were younger than the others. The ratio of Sq from CMUT, CMU1 and JMU were higher, while the ratio of large and small cell carcinoma was higher in Takaoka and CMU1. The lymph node metastasis was most frequent in CMUS, but least in DMU. Tumor size was larger in JMU than DMU. Additionally, the economic level is higher in DMU than JMU, which determines the emphasis of health examination, and subsequently early finding, diagnosis and therapy at early clinicopathological staging. TNM staging was higher in CMUT than JMU. Although both DMU and JMU cities are coastal, where the residents have the high intake of industry-polluted seafood, the residents in JMU smoked the non-filter tobacco, which is closely linked to squamous cell carcinogenesis and male lung cancers. Sq grows slowly, displays large tumor size and male prone, which explains the clinicopathological characteristics of the patients from JMU. Most patients from JMU, especially younger and serious patients, might receive surgical treatment in CMU1 and CMUT, where lung cancer patients showed more aggressiveness than other hospitals, or have the patients’ characteristics of source hospital. The surgotherapeutic levels of Takaoka and CMU1 are higher than the others, where small lung tumors could be diagnosed and dissected for the diagnosis, so that more cases of large and small cell carcinoma are large. CMUS Hospital received too many serious cases from Shenyang and Dalian, which might account for the more diagnosis of lymph node metastasis.

The female patients of lung cancer showed large tumor size, high ratio of Ad and advanced TNM staging. The younger patients of lung cancer displayed small tumor size, a high ratio of Ad, low TNM staging in Takaoka. Tseng et al. demonstrated that the most represented histological type was Ad and the percentages of Ad were 40.2% and 80.4% respectively in males and females with known histology [21]. Fukui's cohort consisted of 1587 patients with Ad (77.0%) and 472 with Sq (23.0%). Female gender, no history of smoking, and small tumor size were distinct characteristics of Ad patients, and a higher age was a characteristic of Sq patients. The younger patients had a higher proportion of Ad, a lower proportion of large cell carcinoma, a higher proportion of stage-I disease and a lower proportion of stage-III disease [22]. A research had showed that NOx and polycyclic aromatic hydrocarbons in the air were risk factors for lung cancer. However, air pollution with nitrates and toxic agents formed from NOx such as nitrosamines might be more important for lung carcinogenesis than polycyclic aromatic hydrocarbons (PAHs) [23]. In animal model, intraperitoneal injection of NNK induced pulmonary Ad in A/J or FVB mice [24], supporting the opinion that air pollution increases the risk of Ad. Furthermore, filter cigarettes has been considered as a cause of Ad and decrease in Sq because the smoke has a lower content of PAHs and low tar, which are thought to be associated with Sq [25]. Inoue et al. [26] found that the younger patients with lung cancer had higher 5-year overall survival rate and lung cancer-related survival rate than the old group, which can be explained by low TNM staging for the younger patients. The proportions of Ad were higher in the young than old groups in agreement with our results [26].

Hong et al. [27] found that there were many risk factors for lung cancer, including tobacco use, environmental pollution, food, genetics, and chronic obstructive pulmonary disease. A meta-analysis of 12 case-control studies also suggested that cooking smoke and second hand smoke were the main risk factors for lung cancer in Chinese women compared with the smoking in male [28]. Females stay home longer time and cook more frequently, housing related exposure has much stronger influence on them than on males. Wang et al. [29] documented that the chrysotile textile workers appeared to have a higher risk of lung cancer than the mining workers at a relatively low exposure level with an interactive effect of asbestos exposure and smoking [30]. Although inhalation of cigarette smoke is a significant risk for lung cancer independent from pack-years [31], Ito et al. [32] found that nicotine dependence, as indicated by the time to first cigarette, is associated with increased risk of lung cancer. Guo et al. [33] found that the increased risks of lung cancer incidence were associated with PM2.5 and ozone air pollution so that air pollution control would be effective for the prevention of lung cancer. Reportedly, the low television watching time, the intake of fruit and Vitamin D, and decreased coffee consumption might be beneficial to the prevention of lung cancer [34–37]. Li et al. [38] found that men with excess body weight had significant decreased chromosome damage levels and lower risk of lung cancer than those with normal-weight. In combination with these findings, we should take advantage of protective factors and avoid the risk factors to prevent lung carcinogenesis, finally to realize the primary prevention of lung cancer.

In conclusion, we found more aggressiveness and shorter survival time of lung cancer in China than Japan. The prevention of lung cancer should be strengthened by establishing a systematic and effective screening strategy, especially for the young and female patients. Although we randomly selected these cases from China and Japan, the limitation of this study was a small number of Japanese lung cancer selected, which might result in analysis bias.

## MATERIALS AND METHODS

### Subjects

The epidemiological data presented in this article were obtained from the inpatient cases of lung cancer from The First Affiliated Hospital (CMU1, n=513), Shengjing Hospital (CMUS, n=1021), Tumor Hospital (CMUT, n=5378) of China Medical University, The First Affiliated Hospital of Dalian (DMU, n=2251) and Jinzhou (JMU, n=630) Medical University, Takaoka Kouseiren Hospital (Takaoka, n=162) of Japan between 2003 and 2013. A detailed personal data and clinical information were collected, including gender, age, site of disease, clinicopathological staging and histological subtyping. However, incomplete clinicopathological data made a smaller number of some parameters in this study. None of these cases received either chemotherapy or radiotherapy before surgery. The Ethical Committees of above-mentioned hospitals approved the research protocol.

### Pathology

The staging for each lung cancer was evaluated according to the TNM system of the Union for International Cancer Control indicating the extent of tumor spread. All cases were confirmed by pathological examination, and so on. Pathological classification was followed by 2004 WHO primary bronchial lung cancer pathological classification criteria, including adenocarcinoma, squamous cell carcinoma, large and small cell carcinomas.

### Statistical analysis

SPSS 10.0 software was employed to analyze all data. Fisher's exact possibility was performed to differentiate the rates and the Mann–Whitney U test to differentiate the means. Kaplan–Meier survival plots were generated and comparisons between the survival curves were made with the log-rank statistic. Cox's proportional hazards model was employed for multivariate analysis. P<0.05 was considered to represent a statistical difference.

## References

[1] GLOBOCAN: International Agency for Research on Cancer (2012). Cancer Incidence and Mortality Worldwide in 2008. http://globocaniarcfr/.

[2] Jemal A, Bray F, Center MM, Ferlay J, Ward E, Forman D (2011). Global cancer statistics. CA Cancer J Clin.

[3] She J, Yang P, Hong Q, Bai C (2013). Lung cancer in China: challenges and interventions. Chest.

[4] Chen W, Zheng R, Zhang S, Zhao P, Zeng H, Zou X, He J (2014). Annual report on status of cancer in China, 2010. Chin J Cancer Res.

[5] Zhou C (2014). Lung cancer molecular epidemiology in China: recent trends. Transl Lung Cancer Res.

[6] Chen W, Zheng R, Zeng H, Zhang S (2015). Epidemiology of lung cancer in China. Thorac Cancer.

[7] Han R, Zheng R, Zhang S, Wu M, Chen W (2013). [Trend analyses on the differences of lung cancer incidence between gender, area and average age in China during 1989-2008]. [Article in Chinese]. Zhongguo Fei Ai Za Zhi.

[8] Devesa SS, Bray F, Vizcaino AP, Parkin DM (2005). International lung cancer trends by histologic type: male: female differences diminishing and adenocarcinoma rates rising. Int J Cancer.

[9] Zou XN, Lin DM, Wan X, Chao A, Feng QF, Dai Z, Yang GH, Lv N (2014). Histological subtypes of lung cancer in Chinese males from 2000 to 2012. Biomed Environ Sci.

[10] Parkin DM, Bray F, Ferlay J, Pisani P (2005). Global cancer statistics, 2002. CA Cancer J Clin.

[11] Pakzad R, Mohammadian-Hafshejani A, Ghoncheh M, Pakzad I, Salehiniya H (2015). The incidence and mortality of lung cancer and their relationship to development in Asia. Transl Lung Cancer Res.

[12] Xu ZY, Blot WJ, Xiao HP, Wu A, Feng YP, Stone BJ, Sun J, Ershow AG, Henderson BE, Fraumeni JF (1989). Smoking, air pollution, and the high rates of lung cancer in Shenyang, China. J Natl Cancer Inst.

[13] Shimada Y, Matsuoka Y (2011). Analysis of indoor PM2. 5 exposure in Asian countries using time use survey. Sci Total Environ.

[14] Hinds MW, Stemmermann GN, Yang HY, Kolonel LN, Lee J, Wegner E (1981). Differences in lung cancer risk from smoking among Japanese, Chinese and Hawaiian women in Hawaii. Int J Cancer.

[15] Osaki Y, Minowa M, Mei J (1999). A comparison of correlates of cigarette smoking behavior between Jiangxi province, China and Japanese high school students. J Epidemiol.

[16] Wang MM, Xiong YN, Zhang SJ, Zhu LH, Liu ZY, Zhang GL (2013). Correlation of Epstein-Barr virus infection and clinical features of patients with lung cancer. Jiangsu Medical J.

[17] Zhang Y, Sun Y, Chen H (2016). Effect of tumor size on prognosis of node-negative lung cancer with sufficient lymph node examination and no disease extension. Onco Targets Ther.

[18] Takahashi Y, Horio H, Hato T, Harada M, Matsutani N, Kawamura M (2015). Predictors of post-recurrence survival in patients with non-small-cell lung cancer initially completely resected. Interact Cardiovasc Thorac Surg.

[19] Yoshida Y, Murayama T, Sato Y, Suzuki Y, Saito H, Tanaka N (2013). Validation of 7th TNM staging system for lung cancer, based on surgical outcomes. Asian Cardiovasc Thorac Ann.

[20] Jiang AG, Chen HL, Lu HY (2015). Comparison of Glasgow prognostic score and prognostic index in patients with advanced non-small cell lung cancer. J Cancer Res Clin Oncol.

[21] Tseng CY, Huang YC, Su SY, Huang JY, Lai CH, Lung CC, Ho CC, Liaw YP (2012). Cell type specificity of female lung cancer associated with sulfur dioxide from air pollutants in Taiwan: an ecological study. BMC Public Health.

[22] Liu M, Cai X, Yu W, Lv C, Fu X (2015). Clinical significance of age at diagnosis among young non-small cell lung cancer patients under 40 years old: a population-based study. Oncotarget.

[23] Franchini M, Mannucci PM (2007). Short-term effects of air pollution on cardiovascular diseases: outcomes and mechanisms. J Thromb Haemost.

[24] Zheng HC, Takano Y (2011). NNK-induced lung tumors: A review of animal model. J Oncol.

[25] Chen F, Cole P, Bina WF (2007). Time trend and geographic patterns of lung adenocarcinoma in the United States, 1973–2002. Cancer Epidemiol Biomarkers Prev.

[26] Inoue M, Okumura M, Sawabata N, Miyaoka E, Asamura H, Yoshino I, Tada H, Fujii Y, Nakanishi Y, Eguchi K, Mori M, Kobayashi H, Yokoi K (2014). Clinicopathological characteristics and surgical results of lung cancer patients aged up to 50 years: the Japanese Lung Cancer Registry Study 2004. Lung Cancer.

[27] Hong QY, Wu GM, Qian GS, Hu CP, Zhou JY, Chen LA, Li WM, Li SY, Wang K, Wang Q, Zhang XJ, Li J, Gong X, Bai CX (2015). Lung Cancer Group of Chinese Thoracic Society; Chinese Alliance Against Lung Cancer. Prevention and management of lung cancer in China. Cancer.

[28] Schuller HM (2002). Mechanisms of smoking-related lung and pancreatic adenocarcinoma development. Nat Rev Cancer.

[29] Wang X, Lin S, Yano E, Yu IT, Courtice M, Lan Y, Christiani DC (2014). Exposure-specific lung cancer risks in Chinese chrysotile textile workers and mining workers. Lung Cancer.

[30] Yano E, Wang X, Wang M, Qiu H, Wang Z (2010). Lung cancer mortality from exposure to chrysotile asbestos and smoking: a case-control study within a cohort in China. Occup Environ Med.

[31] Fukumoto K, Ito H, Matsuo K, Tanaka H, Yokoi K, Tajima K, Takezaki T (2015). Cigarette smoke inhalation and risk of lung cancer: a case-control study in a large Japanese population. Eur J Cancer Prev.

[32] Ito H, Gallus S, Hosono S, Oze I, Fukumoto K, Yatabe Y, Hida T, Mitsudomi T, Negri E, Yokoi K, Tajima K, La Vecchia C, Tanaka H, Matsuo K (2013). Time to first cigarette and lung cancer risk in Japan. Ann Oncol.

[33] Guo Y, Zeng H, Zheng R, Li S, Barnett AG, Zhang S, Zou X, Huxley R, Chen W, Williams G (2016). The association between lung cancer incidence and ambient air pollution in China: A spatiotemporal analysis. Environ Res.

[34] Wakai K, Sugawara Y, Tsuji I, Tamakoshi A, Shimazu T, Matsuo K, Nagata C, Mizoue T, Tanaka K, Inoue M, Tsugane S, Sasazuki S, Research Group for the Development and Evaluation of Cancer Prevention Strategies in Japan (2015). Risk of lung cancer and consumption of vegetables and fruit in Japanese: A pooled analysis of cohort studies in Japan. Cancer Sci.

[35] Ukawa S, Tamakoshi A, Wakai K, Noda H, Ando M, Iso H (2013). Prospective cohort study on television viewing time and incidence of lung cancer: findings from the Japan Collaborative Cohort Study. Cancer Causes Control.

[36] Tang N, Wu Y, Ma J, Wang B, Yu R (2010). Coffee consumption and risk of lung cancer: a meta-analysis. Lung Cancer.

[37] Zhang L, Wang S, Che X, Li X (2015). Vitamin D and lung cancer risk: a comprehensive review and meta-analysis. Cell Physiol Biochem.

[38] Li X, Bai Y, Wang S, Nyamathira SM, Zhang X, Zhang W, Wang T, Deng Q, He M, Zhang X, Wu T, Guo H (2015). Association of body mass index with chromosome damage levels and lung cancer risk among males. Sci Rep.

